# Language and reading impairments are associated with increased prevalence of non‐right‐handedness

**DOI:** 10.1111/cdev.13914

**Published:** 2023-02-13

**Authors:** Filippo Abbondanza, Philip S. Dale, Carol A. Wang, Marianna E. Hayiou‐Thomas, Umar Toseeb, Tanner S. Koomar, Karen G. Wigg, Yu Feng, Kaitlyn M. Price, Elizabeth N. Kerr, Sharon L. Guger, Maureen W. Lovett, Lisa J. Strug, Elsje van Bergen, Conor V. Dolan, J. Bruce Tomblin, Kristina Moll, Gerd Schulte‐Körne, Nina Neuhoff, Andreas Warnke, Simon E. Fisher, Cathy L. Barr, Jacob J. Michaelson, Dorret I. Boomsma, Margaret J. Snowling, Charles Hulme, Andrew J. O. Whitehouse, Craig E. Pennell, Dianne F. Newbury, John Stein, Joel B. Talcott, Dorothy V. M. Bishop, Silvia Paracchini

**Affiliations:** ^1^ School of Medicine University of St Andrews St Andrews UK; ^2^ Department of Speech and Hearing Sciences University of New Mexico Albuquerque New Mexico USA; ^3^ School of Medicine and Public Health University of Newcastle Callaghan New South Wales Australia; ^4^ Department of Psychology University of York York UK; ^5^ Department of Education University of York York UK; ^6^ Department of Psychiatry The University of Iowa Iowa City Iowa USA; ^7^ Division of Experimental and Translational Neuroscience, Krembil Research Institute University Health Network Toronto Ontario Canada; ^8^ Program in Neuroscience and Mental Health Hospital for Sick Children Toronto Ontario Canada; ^9^ Department of Physiology University of Toronto Toronto Ontario Canada; ^10^ Department of Paediatrics University of Toronto Toronto Ontario Canada; ^11^ Department of Psychology Hospital for Sick Children Toronto Ontario Canada; ^12^ Genetics and Genome Biology Hospital for Sick Children Toronto Ontario Canada; ^13^ Dalla Lana School of Public Health University of Toronto Toronto Ontario Canada; ^14^ Department of Biological Psychology Vrije Universiteit Amsterdam Amsterdam The Netherlands; ^15^ Department of Child and Adolescent Psychiatry, Psychotherapy and Psychosomatics Ludwig‐Maximilians‐University Hospital Munich Munchen Germany; ^16^ Julius‐Maximilians‐University Würzburg Würzburg Germany; ^17^ Language and Genetics Department Max Planck Institute for Psycholinguistics Nijmegen The Netherlands; ^18^ Donders Institute for Brain, Cognition and Behaviour Radboud University Nijmegen The Netherlands; ^19^ Department of Experimental Psychology University of Oxford Oxford UK; ^20^ Department of Education University of Oxford Oxford UK; ^21^ Telethon Kids Institute University of Western Australia Perth Western Australia Australia; ^22^ Department of Biological and Medical Sciences Oxford Brookes University Oxford UK; ^23^ Department of Physiology University of Oxford Oxford UK; ^24^ Aston Brain Center, School of Life and Health Sciences Aston University Birmingham UK

## Abstract

Handedness has been studied for association with language‐related disorders because of its link with language hemispheric dominance. No clear pattern has emerged, possibly because of small samples, publication bias, and heterogeneous criteria across studies. Non‐right‐handedness (NRH) frequency was assessed in *N* = 2503 cases with reading and/or language impairment and *N* = 4316 sex‐matched controls identified from 10 distinct cohorts (age range 6–19 years old; European ethnicity) using a priori set criteria. A meta‐analysis (*N*_cases_ = 1994) showed elevated NRH % in individuals with language/reading impairment compared with controls (OR = 1.21, CI = 1.06–1.39, *p* = .01). The association between reading/language impairments and NRH could result from shared pathways underlying brain lateralization, handedness, and cognitive functions.

**Abbreviations**
ALSPC: Avon Longitudinal Study of Parents and Children
CCC: Childrens Communication Checklist
DLD: developmental language disorder
GWAS: Genome‐wide association studies
ICD: International Classification of Disease
KEMH: King Edward Memorial Hospital
LH: left‐handed
NRH: non‐right‐handedness
NTR: Netherlands Twin Register
QHP: Quantitative Hand Preference
RD: reading disability
RH: right‐handedness
SWRT: Single‐Word Reading Test
TOLD‐2P: Test of Language Development 2:P
TROG–2: Test for Reception of Grammar Version 2
WAIT: Wechsler Individual Achievement Test Spelling Test
WISC: Wechsler Intelligence Scale for Children

Handedness is the most obvious lateralized behavioral trait in humans. Most individuals preferentially use one hand versus the other one for most motor tasks, with a strong rightward bias. Across populations, only about 10% of people are left‐handed (LH; Papadatou‐Pastou et al., 2020), with males being ~23% more likely than females to prefer the left hand (Papadatou‐Pastou et al., 2008). Twin studies have estimated the heritability of handedness to be 0.25 (Medland et al., 2009). Hand activities are controlled by the contralateral brain hemisphere such that a right‐ or left‐hand preference implies a left‐ or right‐hemisphere dominance for motor control, respectively (McManus, 2022). The low frequency of left‐handedness across populations has motivated investigations of possible associated disadvantages. A higher rate of non‐right‐handedness (NRH), which includes left‐ and mixed‐handedness, has been reported for neuropsychiatric and neurodevelopmental conditions, such as schizophrenia, autism, and intellectual disability (Hirnstein & Hugdahl, 2014; Markou et al., 2017; Papadatou‐Pastou & Tomprou, 2015). The underlying hypothesis is that the genetic pathways required for establishing left–right brain asymmetries might also contribute to handedness and be involved in neurodevelopmental conditions (Corballis, 2021). It is important to emphasize that the majority of left‐handers are not affected by these conditions and therefore left‐handedness should not be equated to a pathological status.

A link between handedness and language abilities is of particular interest because of the known role of hemispheric lateralization underpinning both traits. Language processing is highly lateralized, involving circuits that reside typically in the left hemisphere, as demonstrated by patients who had language function compromised as the result of strokes affecting the left side of the brain (Kertesz & McCabe, 1977). Left‐handers are more likely than right‐handers to present atypical lateralization for language processing. Specifically, current estimates show that up to 30% of left‐handers present language dominance in the right hemisphere compared with only 5% of right‐handers, with substantial variability across studies (Carey & Johnstone, 2014; Knecht, 2001; Szaflarski et al., 2002; Whitehouse & Bishop, 2009; Woodhead et al., 2021). Such association is more evident in individuals with a very strong left‐hand preference (Mazoyer et al., 2014). Similarly, atypical lateralization for other cognitive domains is more likely to be observed in left‐ than right‐handers (McManus, 2022). Hemispheric dominance for manual praxis (i.e., skilled manual actions) usually resides in the left hemisphere but atypical lateralization has been observed in left‐handers (Vingerhoets, 2019). The pathways involved in and linking different types of asymmetries remain unclear (Fagard, 2013).

The hypothesis that a failure to establish cerebral asymmetries may lead to language disorders was first proposed by Orton who suggested that dyslexia resulted from a failure to establish a complete cerebral dominance (Orton, 1937). Subsequently, the Geschwind‐Galaburda Hypothesis (Galaburda et al., 1985) proposed that reduced hemispheric asymmetries increase the probability of being LH and of developing dyslexia (Galaburda et al., 1985). Annett's Right‐Shift theory also predicted a link between NRH and dyslexia determined by an “asymmetry gene” which would affect the typical left hemisphere lateralization for both language and handedness. This, and other single‐gene theories (McManus, 1985) are not supported by recent genomic studies which show that in most cases, handedness is influenced by the combined effects of variants in a large number of genes (Armour et al., 2014; Cuellar‐Partida et al., 2021; Schmitz et al., 2022). Genome‐wide association studies (GWAS) for handedness have identified some of these genes, some of which have also been implicated in neurodevelopmental conditions, including schizophrenia and dyslexia (Brandler et al., 2013; Brandler & Paracchini, 2014; Cuellar‐Partida et al., 2021; Wiberg et al., 2019).

Language‐associated disorders, including dyslexia (or reading disability, RD) and developmental language disorders (DLD previously referred to as specific language impairment) are reported in about 5%–10% of children, present higher prevalence in males and often co‐occur (Bishop & Snowling, 2004). In both conditions, genetic contributions play a role, with strongest risk factor being an affected first‐degree family member (Arnett et al., 2017; Erbeli et al., 2021; Katusic et al., 2001; Tomblin et al., 1997; Whitehouse, 2010). In twin studies, heritability for both RD and DLD has been reported to be as high as ~70% (Erbeli et al., 2021). Although rare monogenic forms of reading and language disorders have been reported, the majority of cases are polygenic with shared genetic factors contributing to both conditions, as shown by recent and well powered GWASs (Eising et al., 2022; Gialluisi et al., 2014, 2019, 2020). For example, the genetic correlation between single‐word reading (a task used to assess reading abilities) and nonword repetition (a measure of speech perception, phonological short‐term memory and articulation) was reported to be *r* = .7, *p* < .001 (Eising et al., 2022). Genetic studies have also demonstrated complex overlaps between genes contributing to neurodevelopmental disorders, handedness and left/right brain asymmetries. The most recent GWAS for dyslexia conducted in almost 52,000 cases and over 1 million controls reported a significant genetic correlation between dyslexia and ambidexterity (Doust et al., 2021). Genes associated with handedness have been shown to be associated with regional asymmetries of cortical surface areas, including those involved in language‐related circuitry (Sha, Pepe, et al., 2021). A GWAS for brain asymmetry highlighted the role of genes involved in autism and schizophrenia (Sha, Schijven, et al., 2021). Overall, these findings demonstrate with molecular data that brain asymmetries, handedness, and neurodevelopmental disorders, including language‐related conditions, are partially influenced by the same genes. Variants in these shared genes can increase the chances of both being LH and having a neurodevelopmental disorder. The cellular functions associated with the shared genes include cytoskeletal dynamics and the left–right patterning of visceral organs (Paracchini, 2021), supporting the hypotheses that behavioral and anatomical asymmetries might, at least partly, be influenced by the same factors (Brandler & Paracchini, 2014).

At the behavioral level, putative links between handedness and language conditions have been tested both across the normal range of variation observed in the general population as well as in cohorts clinically ascertained for RD or DLD. The literature surrounding a link between handedness and dyslexia is inconsistent, as determined by meta‐analyses (Bishop, 1990; Eglinton & Annett, 1994; Somers et al., 2015). In 1990, Bishop conducted a meta‐analysis of 25 studies examining a total of *N* = 14,159 individuals (Bishop, 1990). Overall, a non‐significant increase of NRH was found in individuals with dyslexia. However, the increase was statistically significant only when the largest study, which had a negative finding and weighted disproportionately on the overall analysis, was omitted. When reanalyzing the complete dataset with a different method, Eglinton and Annett reported a significant over‐representation of NRH among cases with dyslexia (Eglinton & Annett, 1994). In addition to the inconsistency resulting from different analytical methods, Bishop (1990) noted how the heterogeneous criteria used for handedness and dyslexia classification introduced biases in the analyses. For example, studies included in the meta‐analyses measured handedness either as quantitative indexes (Annett & Kilshaw, 1984) or as a category (Felton et al., 1987; Gross et al., 1978). Also, individuals were classified as reading impaired through highly heterogeneous criteria. A recent study compared the epidemiology of dyslexia using both the Statistical Manual in its 5th version (DSM‐5) and the 11th version of the International Classification of Diseases (ICD‐11) on the same sample of 25,000 French pupils. Left‐handedness was associated with dyslexia as defined by the DSM‐5 but not according to the ICD‐11 criteria (Di Folco et al., 2022).

A meta‐analysis for studies investigating potential links between handedness and language abilities found no significant effects in the entire dataset (*N* = 359,890 total individuals; Somers et al., 2015). No differences were detected between males and females. However, analysis in the subgroup of children (age < 16 years) showed a weak handedness effect with right‐handers performing better than non‐right‐handers on verbal skills. High heterogeneity was reported across the studies analyzed reflecting different criteria for group assignment. For example, handedness was assessed in different ways across studies, including self‐reported hand preference for writing (Crow et al., 1998; Gordon & Kravetz, 1991; Kocel, 1977; Peters et al., 2006), different questionnaires (Coulson & Lovett, 2004; Hicks & Beveridge, 1978; Tremblay et al., 2004) and quantitative indexes derived from performance tests like the pegboard task (Annett & Turner, 1974). Inconsistent findings continue to be observed in more recent literature. A small study of 45 individuals with dyslexia and 90 controls found a significant increase of left‐handedness, measured with the Edinburgh Inventory, in the cases (Vlachos et al., 2013). A right‐hand advantage was also reported in a larger study of about 5000 children from the Longitudinal Study of Australian Children (Johnston et al., 2009). LH, and especially mixed‐handed children, tended to perform worse on a broad range of cognitive skills, including reading, writing, and receptive language. This handedness effect was more marked in boys. A similar trend was observed for receptive language, but not for expressive language, implying that NRH‐associated effects might differ between language sub‐domains. Another study with a focus on language abilities found no handedness differences between typically developing (*N* = 156) and children with DLD (*N* = 107; Wilson & Bishop, 2018). In this study, handedness was measured with the Edinburgh Handedness Inventory and the Quantitative Hand Preference (QHP) tasks (see Bishop et al., 1996). The QHP assessment did not show a correlation between handedness and language scores in a general population sample of 569 children (Pritchard et al., 2019).

The inconsistency across results conducted for both reading and language impairment may be due to the different criteria and designs used across studies. Meta‐analyses are a valid approach to extract the most consistent patterns from published studies, although it must be acknowledged that this approach is affected by potential publications biases.

We invited cohorts from the GenLang consortium (https://www.genlang.org/) to participate in this confirmatory study. GenLang is an international collaboration that facilitates large‐scale meta‐analyses in relation to speech, language, reading and related skills. The association between hand preference and language/reading abilities has not been investigated before in these cohorts. Thanks to the availability of raw data, we were able to apply criteria set a priori for defining reading and language impairments to reduce heterogeneity across cohorts. Handedness categories were defined as non‐right (NRH) or right‐handedness (RH) based on the preferred hand for writing or drawing. We report handedness frequency in 10 different cohorts (*N* = 2503 cases with reading and/or language impairment). Eight of these cohorts met the inclusion criteria and entered the meta‐analysis (*N* = 1994 cases).

## MATERIALS AND METHODS

### Study design

This study aims to test whether hand preference is associated with language and reading abilities by comparing the frequency of RH and NRH in cases and controls. We used datasets available through the GenLang Consortium because of their focus on reading and language measures (Table 1). Assignment to case and control groups was based on an existing clinical diagnosis or was derived from psychometric tests (Table S1). In the latter case, assignment to the case group was determined by a score 1 SD or more below the mean on standardized tests for assessing reading or language performance. Participants presenting low scores on performance IQ (i.e., 1 SD below the mean, unless otherwise specified) were excluded to ensure that poor language/reading skills were not secondary manifestations of other neurological or intellectual problems. Children scoring poorly on both language and reading measures were classified as comorbid. Assignment to the control group was based on scores equal to or above the mean of the same reading and language tests, unless otherwise specified. As a result, individuals that scored between the cut‐off criteria for cases and control assignment were excluded from the analysis, ensuring that the controls had no reading or language difficulties. The control groups were individually sex‐matched with the cases to avoid potential bias introduced by the higher prevalence of language disorder and left‐handedness in males. Handedness was defined as the preferred hand for writing and classified as two categories: right‐hand (RH) or non‐right‐hand (NRH) preference. The NRH group included participants who preferred the left‐hand or with no preference (often referred to as ambidextrous). The ambidextrous group was too small to be analyzed separately. This strategy avoided the heterogeneity introduced by the use of different instruments (e.g., different questionnaires or performance test) and classifications (e.g., left/right, right/no‐right, left/mixed/right and left/non‐left) reported in the literature. Controls were not available for three clinical cohorts (UK Dyslexia [UKDYS], Manchester Language Study (MLS) and the Multicenter Study Marburg/Würzburg cohort). For the two UK cohorts, controls were derived from the Avon Longitudinal Study of Parents and Children (ALSPAC) cohort which used directly comparable assessment.

**TABLE 1:** Summary of the cohorts involved in the study.

Cohort	Country	Total *N* ^a^	Cohort type	Phenotype	References
ALSPAC cohort	UK	~13,000	Epidemiological, singletons	Reading, language	Boyd et al. (2013)
Iowa Cohort	USA	~7000	Epidemiological, singletons	Language	Tomblin et al. (1997)
Netherlands Twin Register cohort	Netherlands	~60,000	Epidemiological, twins	Reading	Ligthart et al. (2019)
The Raine Study	Australia	~2900	Epidemiological, singletons	Language	Newnham et al. (1993), Straker et al. (2017)
Twins Early Development Study cohort	England and Wales	~13,000	Epidemiological, twins	Reading, language	Haworth et al. (2013)
Manchester Language Study	UK	~240	Clinical, singletons	Language	Conti‐Ramsden et al. (1997)
Multicenter Study Marburg/Würzburg cohort	Germany	~400	Clinical, singletons and families	Reading	Schulte‐Körne et al. (1996)
Toronto Cohort	Canada	~860	Clinical, families	Reading	Price et al. (2020)
UK Dyslexia Cohort	UK	~1300	Clinical, singletons and families	Reading	Scerri et al. (2017)
York cohort	UK	~260	Clinical, families	Reading, language	Nash et al. (2013)

^a^ Refers to the total number of probands in the initial cohorts.

The third cohort was collected in Germany and could not be matched with suitable controls. This cohort and another (Netherlands Twin Register cohort [NTR]) did not meet the required inclusions and exclusions criteria and therefore were not included in the meta‐analysis. Nevertheless, their handedness frequencies are presented in Table 2. We compared the mean values of possible confounding factors (i.e. performance IQ, total IQ, and birth weight) for the cases stratified by their handedness status (Table S2). We observed no differences for these potential confounders between RH and NRH cases and therefore did not correct our analyses for such factors.

**TABLE 2:** Non‐right‐handedness frequencies.

Cohort name	Cohort type	Phenotype	*N* cases		*N* controls				%NRH	
			NRH (males)	RH	Males %	NRH	RH	Males^e^%	Cases	Controls
				(males)		(males)	(males)			
ALSPAC Language	Epidemiological	Language	27 (15)	214 (127)	.59	112 (69)	749 (450)	.60	.11	.13
ALSPAC Reading	Epidemiological	Reading	30 (22)	168 (101)	.62	112 (69)	749 (450)	.60	.15	.13
IOWA cohort	Epidemiological	Language	22 (16)	182 (105)	.59	56 (35)	610 (360)	.59	.11	.08
NTR cohort^a^	Epidemiological	Reading	31 (18)	203 (97)	.49	136 (66)	914 (450)	.49	.13	.13
The Raine Study	Epidemiological	Language	21 (15)	136 (87)	.65	49 (37)	389 (248)	.65	.13	.11
TEDS Reading	Epidemiological	Reading	29 (8)	163 (84)	.48	143 (60)	1031 (431)	.42	.15	.13
TEDS Language	Epidemiological	Language	34 (11)	187 (75)	.39	143 (60)	1031 (431)	.42	.15	.12
Manchester Language Study^b^	Clinical	Language	34 (28)	133 (103)	.78	93 (69)	586 (450)	.76	.20	.14
Multicenter Study Marburg/Würzburg^c^	Clinical	Reading	22 (19)	255 (189)	.75	NA	NA	NA	.08	NA
Toronto cohort	Clinical	Reading	28 (16)	207 (137)	.65	7 (4)	50 (33)	.65	.12	.12
UKDYS^b^	Clinical	Reading	40 (24)	262 (181)	.68	98 (69)	667 (450)	.68	.13	.13
York Reading	Clinical	Reading	11 (8)	25 (18)	.72	13 (11)	57 (37)	.69	.30	.18
York Language	Clinical	Language	9 (8)	30 (18)	.67	13 (11)	57 (37)	.69	.23	.18
Total			2503			4,316^d^				

*Note*: The table includes the comorbid individuals in the language impairment group.

Abbreviations: ALSPAC, Avon Longitudinal Study of Parents and Children; NA, not available; NRH, non‐right‐handers; RH, right‐handedness; TEDS, Twins Early Development Study; UKDYS, UK Dyslexia.

^a^ This cohort was not included in the meta‐analysis because it lacked IQ data required for group assignment. The NRH frequency is reported.

^b^ These cohorts used overlapping controls from the ALSPAC cohort.

^c^ This cohort was not included in the meta‐analysis because of the lack of comparable controls.

^d^ Refers to the number of unique controls. Overlapping controls were analyzed for the ALSPAC, Manchester Language Study and the UKDYS cohorts.

^e^ Sex‐matching for the ALSPAC, TEDS, and York cohort was done combining the reading and language‐impaired cases.

Overall, this study addresses a long‐standing research question addressing previous limitations, for example, small samples, publication bias and heterogeneity, which affected previous literature.

Given this is a secondary data analysis study, full compliance to the Society for Research in Child Development Sociocultural Policy was not possible.

### Individual cohorts

#### ALSPAC cohort

The ALSPAC is a longitudinal cohort representing the general population living in the Bristol area. The ALSPAC cohort consists of pregnant women in the Avon County, UK, with expected dates of delivery from April 1, 1991 to December 31, 1992 (Boyd et al., 2013; Fraser et al., 2013). The initial number of pregnancies enrolled was 14,541. All children, from age 7, were invited annually for assessments on a wide range of physical, behavioral, and neuropsychological traits, including cognitive (reading ‐and language‐related) measures. Attendance at the annual assessment determined the availability of data for the measures used in this study.

For this study, participants were assigned to the language impairment or reading impairment groups as described previously (Scerri et al., 2011). Briefly, children were excluded if they had (i) a performance IQ score ≤ 85 (Wechsler Intelligence Scale for Children [WISC‐III]; Wechsler et al., 1992), (ii) presence of autistic features based on a Childrens Communication Checklist (CCC) score below −3 SD (Bishop, 1998) (iii) missing data on all relevant phenotypes. Participants were assigned to the reading impairment group when scoring below 1 SD on age‐adjusted single‐word reading at age 7 and age 9 (WORD; Rust et al., 1993). Participants were assigned to the language impairment group when meeting at least two out of four of the following criteria: (i) an overall CCC score below 1 SD from the mean; (ii) an age‐adjusted non‐word repetition score below 1 SD from the mean (Gathercole et al., 1994); (iii) a listening and comprehension test score below 1 SD from the mean (age‐adjusted WOLD; Rust, 1996); (iv) reporting the need for speech/language therapy via a questionnaire. In the case of siblings and twin pairs meeting, the criteria for the impairment group, one child for each nuclear family was selected randomly or based on completeness of the data. Participants were classified as comorbid when meeting the criteria for both reading and language impairments. Assignment to the control group was determined by scores above −0.25 SD from the mean on all the quantitative tests used to assess language and reading impairments as well as no reports of needs for speech/language therapy.

In total, 439 cases (191 language‐impaired, 198 reading impaired, 50 comorbid) and 1138 controls were identified. The control group resulted in 861 individuals after sex‐matching. The cut‐off at −0.25 SD was chosen following a simulation analysis (see Supplementary Material; Table S3) showing that *N* > 1000 controls are necessary to reduce fluctuations in NRH frequency when randomly sex‐matching (*N*_simulation_ = 1000). Setting the cut‐off above the mean of all tests would have resulted in a smaller sample (*N* = 592), leading to larger fluctuations of NRH. We also used a simulation to test for potential biases introduced by the use of a single set of controls for comparing both the reading and language impairment groups. No inflation was detected (Supplementary Methods). The same observation applies to the Twins Early Development Study (TEDS) and York cohorts.

Handedness was assessed as the self‐reported preferred hand for writing at age 7 and coded as a binary variable (“Right” or “Left”). The study website contains details of all the data through a fully searchable data dictionary (http://www.bristol.ac.uk/alspac/researchers/our‐data/).

Ethical approval for the study was obtained from the ALSPAC Ethics and Law Committee and the local research ethics committees (http://www.bristol.ac.uk/alspac/researchers/research‐ethics/). Informed consent for the use of data collected via questionnaires and clinics was obtained from participants following the recommendations of the ALSPAC Ethics and Law Committee at the time.

#### Iowa cohort

The Iowa cohort is a cross‐sectional epidemiological study of early language ability in 5‐ and 6‐year‐old children (Tomblin et al., 1997). A total of 7218 children were screened for language ability with a 40‐item subset of the Test of Language Development 2:P (TOLD‐2P; Newcomer & Hammill, 1988). Inclusion criteria for entering the study included being monolingual English speakers without hearing loss. The 26.2% of children who failed the test during the language ability screening were selected to compose approximately half of the final cohort. The other half was randomly selected from the children who passed the screening test. In total, the cohort included 1929 children. A more comprehensive battery of language assessments—consisting of the five principal subtests of the TOLD‐2P, and a discourse task with both narrative comprehension and production components (Culatta et al., 1983)—was used to derive a composite language score (age 5–6). For this study, participants were excluded if they had a performance IQ score below 85 (WISC‐IV; Wechsler, 2012). Participants scoring below 1 SD and above the mean on the composite score were assigned to the case and control group, respectively. A total of 204 cases and 666 sex‐matched controls were selected. Handedness was defined as left‐ or right‐hand used to draw a picture, as assessed by the child's examiner.

Analysis of the Iowa cohort was approved under the University of Iowa IRB #201406727, which covers secondary data analysis of the data originally collected under the University of Iowa IRB #200511767 under which all subjects (or legal guardians) provided informed consent/assent, as appropriate.

#### NTR cohort

The NTR is a national register including more than 120,000 twins and their relatives (Ligthart et al., 2019). The twins were assessed repeatedly using a range of cognitive and behavioral tasks at regular intervals. Teachers provided test scores on the nationally standardized tests that form the Dutch Pupil Monitoring System. Reading ability (or decoding fluency) was assessed with a single‐word reading test by asking children to read aloud as many words as possible from a word list within 1 min. Children were tested at school in Grades 1–6, with up to three word‐reading fluency lists, administered by the teacher to children individually (Verhoeven, 1995; Verhoeven & van Leeuwe, 2009).

For the current study, the score at the latest measurement was used. Children were excluded for (i) not attending mainstream education programs, or (ii) missing data. Participants were defined as cases if they scored in the bottom 10th percentile based on the national norms in Dutch education (equivalent to 1.28 SD below the mean), which was the closest cut‐off that could be applied to conform to our criteria. Individuals scoring above the mean of the national norms were assigned to the control group. A total of 234 individuals with reading impairment and 1050 sex‐matched controls were selected. Because of the lack of IQ data, this cohort was not included in the meta‐analyses.

Handedness was recorded in questionnaires for the mothers as hand preference for “drawing on a piece of paper” at age 5. Answer options were right‐, left‐ or no‐preference. The left‐ and no‐preference were merged in the NRH category.

Ethical approval was granted by the Vrije Universiteit Amsterdam's Medical Ethics Committee (NTR/25‐05‐2007). Data were collected following parental consent.

#### The Raine Study

The Raine Study is a prospective pregnancy cohort that recruited 2900 women between 1989 and 1991 (Newnham et al., 1993; Straker et al., 2017). Recruitment took place at Western Australia's major perinatal center, King Edward Memorial Hospital (KEMH), and nearby private practices.

The mothers (Gen1) completed questionnaires regarding their children (Gen2) who underwent physical examinations at ages 1, 2, 3, 6, 8, 10, 14, 17, 20, and 22 years. The data used for this study were from the assessment at 10 years of age. Participants were excluded if they had a performance IQ score below 1 SD from the mean assessed through the Raven Coloured Progressive Matrices test (Raven et al., 1996). Total standard scores of the CELF‐3 (Semel et al., 1995) were used for group assignments. Participants were assigned to the case group when scoring equal or below 1 SD from the mean, and to the control group when scoring above the mean. This resulted in *N* = 157 language‐impaired cases and *N* = 438 sex‐matched controls. Hand preference for writing was self‐reported and recorded in the McCarron Assessment of Neuromuscular Development (McCarron, 1997).

The study was approved by the Human Ethics Committee at KEMH, Princess Margaret Hospital for Children, the University of Western Australia and the Health Department of Western Australia.

#### TEDS cohort

The TEDS is a longitudinal study of a cohort of twins from over 13,000 families born in England and Wales between 1994 and 1996 (Haworth et al., 2013; Rimfeld et al., 2019). The cohort includes a broad range of phenotypic data, including language and reading skills and handedness. The TEDS website includes a complete data dictionary https://www.teds.ac.uk/datadictionary/home.html, which details exclusions based on medical and perinatal factors, missing data, and other factors. For this study, participants were excluded if they had a performance IQ score that was below 1 SD based on Raven Matrices and Picture Completion tests. Individuals were assigned to the language impairment group when scoring 1 SD below a language composite score mean (Hayiou‐Thomas et al., 2021). Briefly, the composite score was based on a battery of audio‐streamed, web‐administered measures including vocabulary (WISC‐III‐PI; Kaplan, 1999), syntax (Listening Grammar; Test of Adolescent & Adult Language‐3; Hammill et al., 1994), non‐literal semantics, and understanding of inferences (Test of Language Competence‐Level 2; Wiig & Secord, 1985) administered at age 12. Previous analysis showed substantial phenotypic and genetic overlap among these four measures (Dale et al., 2010). The four tests were standardized and averaged.

Participants were assigned to the reading impairment group if they scored 1 SD below the mean of a reading fluency composite score (Hayiou‐Thomas et al., 2021). Briefly, children completed an online adaptation of the Woodcock‐Johnson III Reading Fluency test (Woodcock et al., 2001). In addition, the Test of Word Reading Efficiency (TOWRE Form B; Torgesen et al., 1999) was included in a test booklet sent to families by mail and administered to each twin separately by telephone. Previous work with the TEDS sample established strong concurrent validity for telephone administration of the TOWRE (Dale et al., 2005). The tests were standardized and averaged.

Participants scoring 1 SD below the mean for both the language and reading composite scores were assigned to the comorbid group. Participants scoring above −0.25 SD from the mean of both composite tests were assigned to the control group. One child per twin pair was selected at random if both twins had the relevant phenotypes. A total of 413 cases (*N* = 192 cases with reading impairment; *N* = 152 cases with language impairment, *N* = 69 comorbid) and 1174 sex‐matched controls were identified.

The primary measure of handedness was self‐reported at 16 years. It included a question asking the preferred hand used for writing (left, right, mixed). The TEDS study received ethical approval from the King's College London Ethics Committee.

#### Manchester Language Study cohort

The MLS followed 242 children with language impairment (Conti‐Ramsden et al., 1997). Probands were recruited at age 7 from 118 language units attached to English mainstream schools (Conti‐Ramsden et al., 1997; Conti‐Ramsden & Botting, 1999). Participants were contacted and reassessed again at ages 8 (*N* = 232), 11 (*N* = 200), 14 (*N* = 113), 16 (*N* = 139), and 24 (*N* = 84) years old. All children attended a language unit for at least 50% of the week, and as such, met the criteria for a language impairment diagnosis. Children with other neurological difficulties, hearing impairment, a diagnosis of autism or a general learning disability were excluded. Participants were excluded when they had a Raven matrices performance score IQ that was more than 1 SD below the mean. A total of 167 cases were selected for the current study. Handedness was assessed at age 8 as self‐reported hand preference (“are you left‐ or right‐handed?”). If data were not available at age 8 (*N* = 26), reports from age 14 were used. Hand preference was consistent in 97% of the participants who had data at both time points. Controls were not available for the MLS cohort, and therefore were derived from the ALSPAC control group resulting in *N* = 679 after sex‐matching.

Ethical approval was given by The University of Manchester Research Ethics Committee, UK. Parents or legal guardians provided informed consent for all participants up to the age of 16 years.

#### Multicenter study Marburg/Würzburg cohort

The Marburg/Würzburg cohort is a family‐based cohort that focuses on the genetic basis of reading impairment (Schulte‐Körne et al., 1996). Participants were excluded if they had (i) Nonverbal IQ < 85 (Culture Fair Intelligence Test; Weiß, 1998), (ii) presence of visual or auditory impairments, (iii) inadequate schooling and absences for more than 6 weeks per school year, (iv) first language other than German, (v) diagnosis of attention deficit hyperactivity disorder (ADHD), and (vi) presence of psychiatric disorders, seizure disorder, and use of medication affecting the central nervous system. The study enrolled 403 probands between 8 and 19 years old (grades 2 to 11). Probands were assessed on a large cognitive battery including reading and arithmetic skills, and neurophysiological correlates (ERP studies) associated with language and reading processing. Of the 403 participants, 277 scored more than 1 SD below the mean on single‐word reading (see Schulte‐Körne et al., 1996) meeting the criteria for assignment to the reading impairment group. Handedness was measured by a questionnaire including 10 items describing a specific activity (e.g., writing, throwing a ball, brushing teeth). Participants reported which hand they used for the specific activity based on a four‐point rating scale (1 = always left, 2 = mostly left, 3 = mostly right, 4 = always right). For the current study, only hand preference for writing was considered. Answers 1 and 2 were coded as “non‐right” and answers 3 and 4 were coded as “right.” No controls assessed with comparable measures were available, and therefore, this cohort was not included in the meta‐analyses. Ethical approval was obtained from the ethics committees of the Universities of Marburg and Würzburg.

#### Toronto cohort

Children between the ages of 6 and 16 years who struggled primarily with reading acquisition were recruited from the Toronto area and across Ontario (Couto et al., 2008; Elbert et al., 2011; Price et al., 2020; Tran et al., 2014). Siblings in the same age range with or without reading difficulties were also invited to participate.

Individuals were excluded for a performance IQ < 80 (WISC‐III) on either Verbal Comprehension or Perceptual Reasoning on the WISC‐IV. Three main reading subtests were used to determine reading impairment: (i) Word Identification and (ii) Word Attack from the Woodcock Reading Mastery Tests Revised (Woodcock, 1987) and (iii) Reading subtest of the Wide Range Achievement Test (WRAT‐3; Wilkinson, 1993) Individuals were assigned to the reading impairment group if they scored at least 1.5 SD below the mean on 2 out of 3 reading measures or at least 1 SD below the mean on all three measures. Controls were defined as scoring above the mean on all three measures. A total of 235 cases and 57 sex‐matched controls were included in the analyses. If families included multiple children meeting these criteria, one child was selected at random.

Right‐ and left‐hand preference was determined by a psychometrist as the child wrote to complete the WISC‐IV Coding test. The participants provided verbal or written consent and the parents provided written consent. The study was approved by the Hospital for Sick Children and University Health Network Research Ethics Boards.

#### UKDYS cohort

The UKDYS cohort includes nuclear families and singletons recruited to study the genetics of dyslexia (Scerri et al., 2010, 2017). The family cohort was recruited by research clinics in Oxford and Reading and included 689 siblings from 409 families. The singleton cohort was recruited in clinics in Oxford, Reading and Aston, and included 676 children. The age at assessment ranged from 7 years to 18 years.

For this study, individuals were excluded when presenting performance IQ scores <85 (WISC‐III) and were assigned to the case group if they scored at least 1 SD below the mean on the British Abilities Scales single‐word reading test (Thomson, 1982). Handedness was defined as self‐reported hand preference for writing (“Right” or “Left”). In total, 302 children met the criteria for reading impairment. Controls were derived from the main ALSPAC control group (*N* = 765 sex‐matched controls).

Ethical approvals for the Oxford family and case/control cohorts were granted by the Oxfordshire Psychiatric Research Ethics Committee (OPREC O01.02). Ethical approval for the Aston cohort was granted by the Aston University Ethics Committee.

#### York cohort

The York cohort comprises 260 children who were followed longitudinally in a study of language and reading disorders (Nash et al., 2013). Children were assessed on a battery of cognitive, language, and reading tests approximately annually between the ages of 3½ and 9 years. Assignment to the reading and language impairment group was based on the assessment at age 8–9 years old (described fully in Snowling et al., 2019). Children with performance IQ < 85 (WISC‐IV) were excluded. For this study, a reading impairment outcome was defined based on a score 1 SD or more below the mean, on a reading composite measure of the Single‐Word Reading Test (SWRT 6–16; Foster, 2007) and the Wechsler Individual Achievement Test Spelling Test (WIAT–II; Wechsler, 2005). A language impairment outcome was defined based on a score 1 SD or more below the mean, on a composite language measure of Expressive Vocabulary (CELF–4 UK; Wiig et al., 2006), Test for Reception of Grammar Version 2 (TROG–2; Bishop, 2003), and Formulated Sentences (CELF–4). According to these criteria, 36 children had reading impairment, 20 children had language impairment, and 19 children showed comorbidity for both conditions. Participants scoring above the mean for both the reading and language composite scores were sex‐matched to the combined cases, resulting in *N* = 70 controls. Handedness was defined as self‐reported hand preference for writing collected at age 8 years as “Right” or “Left” categories.

Ethical approval for the study was provided by the University of York, Department of Psychology's Ethics Committee and the NHS Research Ethics Committee. Parents provided written informed consent for their child to be involved.

### Statistical analyses

Handedness frequency was compared between cases and controls using random‐effect meta‐analyses with and without moderators for impairment type (language/reading impairment) and cohort type (clinical/epidemiological). The number of individuals with comorbidities was too small to be analyzed separately and was therefore combined with the language impairment group. The analysis was also run including individuals with comorbidities in the reading impairment group (see Supplementary Material).

Meta‐analyses were conducted using the *rma* function in the R package *metafor* (test = “knha,” Balduzzi et al., 2019; R Core Team, 2019) under REML random effect model. The presence of heterogeneity between groups was explored using the Cochran's *Q* test and the *I* ^2^ index. The summary data for all cohorts and the code to run the analysis are available at https://github.com/fabbondanza/GenLang_hand_preference_meta_analysis.

## RESULTS

We investigated the frequency of NRH in individuals with reading or language impairment (*N* total cases = 2503) from 10 cohorts (Table 2). Overall, the NRH frequency ranged from 8% in the Multicenter Study Marburg/Würzburg cohort (*N*_cases_ = 277) to 30% in the York reading cohort (*N*_cases_ = 36). In the controls, NRH ranged from 8% (IOWA, *N*_controls_ = 666) to 18% (York, *N*_controls_ = 70). When excluding the York cohort, which appeared to be an outlier for both cases and controls and had a small sample size, the NRH frequency ranged from 8%–20% in the cases and 8%–14% in the controls.

### Meta‐analysis

The NTR cohort and the Multicenter Study Marburg/Würzburg cohort were excluded from the meta‐analysis because of the lack of IQ data or suitable controls, respectively. We meta‐analyzed data from 8 cohorts, including 4 clinical and 4 epidemiological cohorts.

**FIGURE 1:**
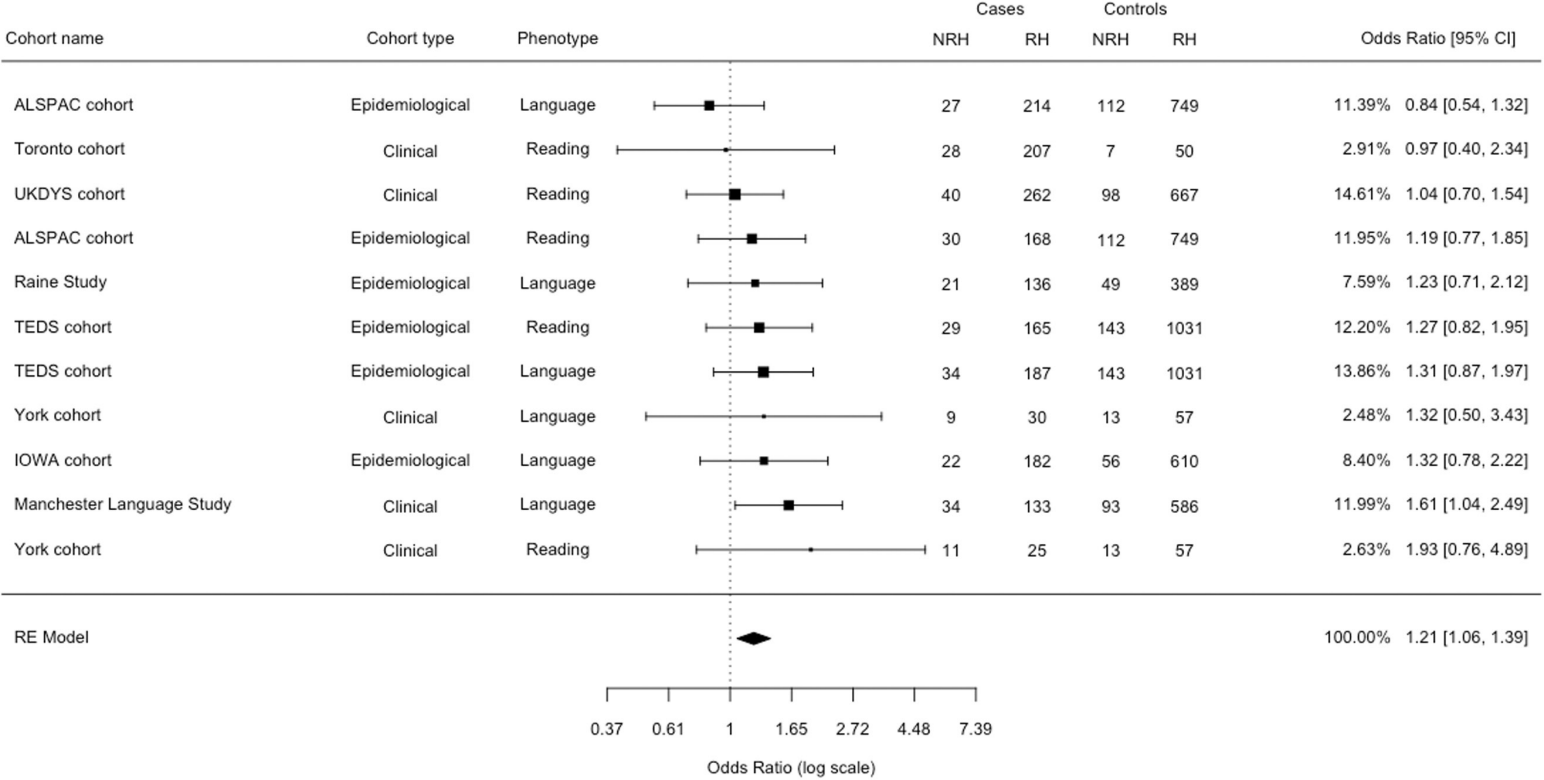
Meta‐analysis of non‐right‐handedness (NRH) frequency in individuals with language/reading impairments. The forest plot shows the results of the meta‐analysis run under a random effect (RE) model. The OR estimates are shown with the 95% confidence interval and the weights (in percentages) on the overall result of OR = 1.21, CI = 1.06–1.39 (*t* = 3.16, *p* = .01). See Figure S1 for the corresponding funnel plot. ALSPAC, Avon Longitudinal Study of Parents and Children; RH, right‐handedness; TEDS, Twins Early Development Study; UKDYS, UK Dyslexia.

We observed an increase of NRH in the case group (OR = 1.21, CI = 1.06–1.39, *t* = 3.16, *p* = .01; Figure 1). Egger's t test showed no evidence of funnel plot asymmetry (*t* = 0.563, *p* = .59, df = 9). We observed no evidence of heterogeneity (*Q* (10) = 6.27, *p* = .79, *τ* ^2^ = .01, *I* ^2^ = 0%; see Figure S1 for funnel plot). When the MLS and UKDys which lacked independent controls were removed, the results remained comparable to those observed in the full datasets (OR = 1.19, CI = 1.03–1.38, *t* = 2.78, *p* = .02, Figure S2). Inclusion of the comorbid individuals as part of the reading impairment group made no major difference (Figures S3 and S4). The lowest OR (0.84) was observed for an epidemiological cohort, while the highest ORs (1.93) were observed in clinical cohorts. However, a formal analysis did not reveal a moderator effect of cohort type (clinical vs. epidemiological; *p* = .21) or type of impairment (reading vs. language; *p* = .59).

## DISCUSSION

We investigated the prevalence of NRH in individuals with reading and language impairments in a total of 2503 cases from 10 cohorts. NRH frequency tended to be elevated and presented a wider range of variation in the cases (8%–30%) compared with the controls (8%–18%). The upper range of variation was observed in the York cohort for both cases and controls, possibly suggesting a bias introduced by how NRH was assessed. However, the NRH prevalence in this cohort could have also been inflated random variations associated with the small sample size and the high rate of males. The second‐highest level of NRH was observed in the MLS (20%), a clinical cohort collected for language impairment. The high rate of NRH likely reflects a genuine association with a particularly severe language phenotype considering that the MLS was recruited following very stringent inclusion criteria. The lowest level of NRH in cases (8%) was observed in the Multicenter Study Marburg/Würzburg cohorts which lacked internal controls and therefore could not be evaluated for a potential assessment bias.

The meta‐analysis was conducted in the eight cohorts that met the inclusion criteria (*N*_cases_ = 1994). Overall, we observed a higher rate of NRH in individuals with language/reading impairment compared with controls (OR = 1.21, CI = 1.06–1.39, *t* = 3.16, *p* = .01). The availability of raw data allowed us to apply similar criteria for group definition, yet it is worth noting that all cohorts analyzed here were originally recruited for different types of studies and designs. Nevertheless, no moderator effects were detected for impairment (reading vs. language) or cohort type (epidemiological vs. clinical). No changes in the results were observed when the comorbid groups, which were too small to be analyzed individually, were included in either the language or the reading impaired groups. A similar but attenuated trend was observed after removing the UKDYS and Manchester Language cohorts, thus ruling out a possible bias introduced by the lack of independent controls. The removal of the York cohort, which represented an outlier, also led to a similar but attenuated trend (OR = 1.19, CI = 1.03–1.38, *p* = .02; Figure S5). Although we analyzed almost 2000 cases, the sample sizes of the individual cohorts were too small to test subgroups selected for phenotype severity or disorder subtype, when considering the small effect size observed in the whole sample. A systematic assessment of handedness in larger cohorts of individuals, recruited and assessed with the same criteria for reading or language impairment, will be necessary to differentiate potential group‐specific effects and to evaluate differences between clinical and population‐based cohorts.

We acknowledge that the use of overlapping controls derived from the ALSPAC cohort and used for the UKDYS and Manchester Language cohort is not ideal, as non‐independent datasets might lead to biases (Noble et al., 2017). When the UKDYS and the Manchester Language cohorts were excluded, the results were comparable (same direction, but attenuated strength; OR = 1.19, CI = 1.03–1.38, *p* = .02) to the full dataset. An alternative option could have been the use of non‐overlapping controls from ALSPAC. However, a simulation analysis showed that the use of smaller subsets of independent controls would increase the fluctuation of NRH and thus increase the noise in the analysis. Cultural factors, such as stigma against left‐handedness, are known to vary to some extent with ethnicity and generations, but this is not a concern for our study. The children analyzed have similar birth years and large studies in the UK Biobank have not identified geographical factors that influence handedness prevalence within England (de Kovel et al., 2019). However, it is worth noting that our study is limited to cohorts of White European ancestry and therefore generalizability of our results will require analysis in other populations.

Previous meta‐analyses of the literature have been inconclusive (Bishop, 1990; Eglinton & Annett, 1994; Somers et al., 2015) and studies that applied different definitions of dyslexia found inconsistent results in the same dataset (Di Folco et al., 2022). When applying the DSM‐5, which is more closely in line with the criteria adopted here, Di Folco and colleagues found an association between handedness and dyslexia that was very similar to our study (OR = 1.24, *p* = .003). The effect disappeared when applying the ICD‐11 definition which is based on IQ discrepancy. Di Folco and colleagues concluded that the original effect was not specific to reading but mediated by IQ. Such a conclusion was supported by the observation that “non‐right‐handers scored on average 2 IQ points lower than right‐handers.” When comparing IQ between NRH and RH cases in the present study, we observed no significant differences with the exception of the UKDYS cohort (uncorrected *p* = .03). Our observation is in line with meta‐analyses investigating the associations between handedness and cognitive abilities, which reported that right‐handers had only marginally higher scores compared with left‐handers (Ntolka & Papadatou‐Pastou, 2017).

Some potential issues affecting the reliability of our data could have been introduced by the assessment of handedness at a young age. Hand preference can fluctuate in the early years of development but is well established by the time a child is 3 years old (McManus et al., 1988). In all our cohorts, handedness data were collected when children were at least 5 years old, and therefore after the handedness direction is fully established, as demonstrated also by the high correlation of assessments conducted at different time points (e.g., ALSPAC: *r* = .95 CI = [0.93, 0.97], *p* < 2.2 × 10^−16^, Schmitz et al., 2022).

In summary, our study investigates an old question with new data addressing issues that affected the previous literature, including small samples, heterogeneous criteria, and publication bias. The findings support an association, albeit small in size, between NRH and language/reading impairment, expanding the range of neurodevelopmental traits (e.g., autism and schizophrenia) known to be associated with handedness. From these data, it is not possible to infer any cause/effect directionality between brain asymmetries, disorders, and handedness but provide an important foundation for theoretical framework. Our results are in line with the evidence emerging from genetic studies supporting the role of shared genes and biological pathways contributing to both lateralization and neurodevelopmental disorders.

## FUNDING INFORMATION

Silvia Paracchini and Filippo Abbondanza are funded by the Royal Society (UF150663; RGF\EA\180141). The UK Medical Research Council and Wellcome (Grant ref: 217065/Z/19/Z) and the University of Bristol provide core support for ALSPAC. This publication is the work of the authors and will serve as guarantors for the contents of this paper. A comprehensive list of grants funding is available on the ALSPAC website: http://www.bristol.ac.uk/alspac/external/documents/grant‐acknowledgements.pdf. The funders had no role in study design, data collection and analysis, decision to publish, or preparation of the manuscript. Elsje van Bergen was supported by NWO VENI fellowship 451‐15‐017. Support for the Toronto cohort collection was provided by grants from the Canadian Institutes of Health Research (MOP‐133440). K.M.P. was supported by the Hospital for Sick Children Research Training Program (Restracomp). Simon Fisher is funded by the Max Planck Society. Dorothy Bishop is funded by European Research Council Advanced Grant 694189. Andrew Whitehouse is supported by an Investigator Grant from the National Health and Medical Research Council (1173896). The Raine Study was supported by the National Health and Medical Research Council of Australia (grant numbers 572613, 403981, 1059711), and the Canadian Institutes of Health Research (grant number MOP‐82893). The Multicenter Study Marburg/Würzburg cohort was funded by the Deutsche Forschungsgemeinschaft (DFG).

## Supporting information


Appendix S1.
