# Supplementary material for: Language and reading impairments are associated with increased prevalence of non‐right‐handedness
Source: Child Dev. 2023 Feb 13;94(4):970–84. doi: 10.1111/cdev.13914 (PMC10330064; doi:10.1111/cdev.13914)
Supplement: Supplementary file 1 — Appendix S1. [file CDEV-94-970-s001.docx]

**Supplementary Information**

**Language and reading impairments are associated with increased prevalence of non-right handedness**

**Supplementary Methods**

**Simulations**

The ALSPAC control group was used as the control group for the Manchester Language Study and the UK Dyslexia cohort after a random sex-matching process. Therefore, we ran a simulation analysis to evaluate how variation in the size of the initial sample would affect non-right handedness (NRH) frequency after sex-matching.

We calculated the lowest and highest NRH frequencies when extracting a sample of 500, 750 and 1,000 random controls (N_simulation_ = 1,000; Supplementary Table S3). The simulation showed that in a sample of 500 controls NRH would range between 8% and 15%. This range was reduced to 11%-13% for a sample of 1000 individuals. As a result, we used a cut-off of -0.25 SD above the mean of all tests, resulting in a starting sample of N = 1,138 initial controls. For consistency, we applied the same cut-off to the TEDS controls.

In three cohorts (ALSPAC, TEDs and York) it was possible to identify individuals who had either reading or language impairment (or both). The two case groups (reading and language impairment) were compared to a single control sample. We ran a simulation to test whether the use of the same controls for comparisons with two different sets of cases could inflate the results. We created synthetic controls for all cohorts and ran the meta-analysis (N_simulation_ = 1000). No inflation of false positives was highlighted (47 out of 1000 runs were significant).

**Supplementary Tables**

**Supplementary Table S1: Inclusion and exclusion criteria**

| **Cohort** | **Exclusion criteria** | **Case definition** | **Control definition** | **Reference for tasks used** |
| --- | --- | --- | --- | --- |
| **ALSPAC Language** | Performance IQ  WISC-III <- 85 | Two of the following:  i) CCC < -1SD  ii) WOLD < -1 SD  iii) NWR < -1SD  iv) reporting need of speech/language therapy via a questionnaire | CCC > -0.25 SD  WOLD > -0.25 SD  NWR > -0.25  Not reporting need of speech/language therapy via a questionnaire | (Wechsler, Golombok, & Rust, 1992)   (Bishop, 1998)  (Rust, 1996)  (Gathercole, Willis, Baddeley, & Emslie, 1994)  (Rust, Golombok, & Trickey, 1993) |
| **ALSPAC Reading** |  | WORD age 7 < -1SD  WORD age 9 < -1SD |  |  |
| **IOWA** | Performance IQ  WISC-IV < 85 | TOLD < -1 SD | TOLD > 0 | (Wechsler, 2012)  (Culatta, Page, & Ellis, 1983) |
| **NTR** |  | National score < -1.28 SD |  |  |
| **The Raine Study** | Performance IQ  Raven Matrices < 85 | CELF < -1 SD | CELF > 0 | (Raven, Court, & Raven, 1996)  (Semel, Wiig, & Secord, 1995) |
| **TEDS Reading** | Performance IQ  Raven Matrices < 85 | CRS < -1 SD | CRS > -0.25 SD  CLS > -0.25 SD | (Raven et al., 1996)  (Hayiou-Thomas, Smith-Woolley, & Dale, 2021) |
| **TEDS Language** |  | CLS < -1 SD |  |  |
| **Manchester Language Study^2^** | Performance IQ  Raven matrices < 85 | Language assessment | / | (Raven et al., 1996)  (Conti-Ramsden, Crutchley, & Botting, 1997) |
| **Multicenter Study Marburg/Würzburg^3^** | Performance IQ  Culture Fair Intelligence Test < 85 | SWRT < -1 SD | / | (Weiß RH, 1998)  (Schulte-Körne, Deimel, Müller, Gutenbrunner, & Remschmidt, 1996) |
| **Toronto** | Performance IQ  WISC-III < 80 | Scores < -1.5 SD for 2 out of 3 reading tests;  or scores < -1 SD on average of all three tests  i) WRMT, Word identification  ii) WRMT, Word Attack  iii) WRAT-3 | > 0 for all reading tests | (Wechsler et al., 1992)  (Woodcock, 1987)  (Wilkinson, 1993) |
| **UKDYS** | Performance IQ  WISC-III < 85 | WR (BAS) < -1 SD | / | (Wechsler et al., 1992)  (Thomson, 1982) |
| **York Reading** | Performance IQ  WISC-IV < 85 | CRS (SWRT; WIAT) < -1 SD | CRS > 0  CRL > 0 | (Wechsler, 2012)  (Foster, 2007)  (Wechsler, 2005)  (Semel, Wiig, & Secord, 1992) |
| **York Language** |  | CLS (CELF, TROG) < -1 SD |  | (Bishop, 2003) |

The inclusion and exclusion criteria refer to this study. For criteria defining the design of the original cohorts see the main manuscript.

Abbreviations; WISC, Wechsler Intelligence Scale for Children; CCC Children Communication Checklist; WOLD, Wechsler Objective Language Dimensions; NWR, non-word repetition; TOLD, Test of Language Development; CELF, Clinical Evaluation of Language Fundamentals; CRS, Composite Reading Score; CLS, Composite Language Score; WRMT, Woodcock Reading Mastery Tests; WRAT, Wide Range Achievement Test; WR, World Reading; BAS, British Abilities Scales; SWRT Single Word Reading Test; WIAT, Wechsler Individual Achievement Test Spelling Test;

**Table S2: comparison of IQ and birth weight mean values between NRH and RH cases**

| **Cohort** | **Cohort type** | **Phenotype** | **Total IQ (SD)** | | **Performance IQ (SD)** | | **Birth weight (gr; SD)** | |
| --- | --- | --- | --- | --- | --- | --- | --- | --- |
|  |  |  | **NRH** | **RH** | **NRH** | **RH** | **NRH** | **RH** |
| **ALSPAC Language** | Epidemiological | Language | 106 (13.8) | 101 (12.8) | 107 (12.9) | 102(12.8) | 3479 (477) | 3426 (521) |
| **ALSPAC Reading** | Epidemiological | Reading | 97.9 (11.0) | 99 (10.6) | 99.5 (11.4) | 101 (11.5) | 3434 (613) | 3425 (521) |
| **IOWA** | Epidemiological | Language | / | / | 99.6 (8.2) | 97.9 (8.6) | / | / |
| **NTR** | Epidemiological | Reading | / | / | / | / | 2422 (647) | 2412 (588) |
| **The Raine Study** | Epidemiological | Language | / | / | 45.43 (25.78) | 45.96 (20.97) | 3473 (579.77) | 3329 (641.19) |
| **TEDS Reading^1^** | Epidemiological | Reading | 0.136 (0.810) | 0.0741(0.678) | 0.246 (0.622) | 0.211 (0.717) | 2612 (461)* | 2391 (525)* |
| **TEDS Language^1^** | Epidemiological | Language | -0.898 (0.623) | -0.777 (0.574) | -0.207 (0.583) | -0.115 (0.633) | 2319 (513) | 2507 (503) |
| **Manchester Language Study** | Clinical | Language | / | / | 108.7 (11) | 110 (12)) | 3401 (640) | 3347 (544) |
| **Multicenter Study Marburg/Würzburg** | Clinical | Reading | / | / | 107.4 (12.1) | 109.3 (12.7) | / | / |
| **Toronto** | Clinical | Reading | 98.42 (7.87) | 95.08 (9.03) | 104.33 (8.74) | 101.38 (9.07) | 3409 (679) | 3553 (553) |
| **UKDYS^1^** | Clinical | Reading | / | / | 0.16 (0.57)* | 0.42 (0.75)* | / | / |
| **York Reading** | Clinical | Reading | / | / | 97 (9.39) | 102 (10.6) | / | / |
| **York Language** | Clinical | Language | / | / | 83.3 (14.3) | 89.7 (14.1) | / | / |

NA = not available; NRH = Non-right handers cases; RH = Right handers cases.

See Supplementary Table S1 for details on the tests used for IQ assessment.

Comorbid individuals have been assigned to language impairment group.

*Significant difference between NRH and RH cases; t-test, *p* < 0.05 uncorrected for multiple testing

^1^ IQ data presented as standardised scores

**Supplementary Table S3. Simulation analysis of NRH frequency**

| **Starting sample size** | **Min NRH %** | **Max NRH f%** |
| --- | --- | --- |
| 500 | 0.08 | 0.15 |
| 750 | 0.09 | 0.14 |
| 1000 | 0.11 | 0.13 |

**Supplementary Figures**

**
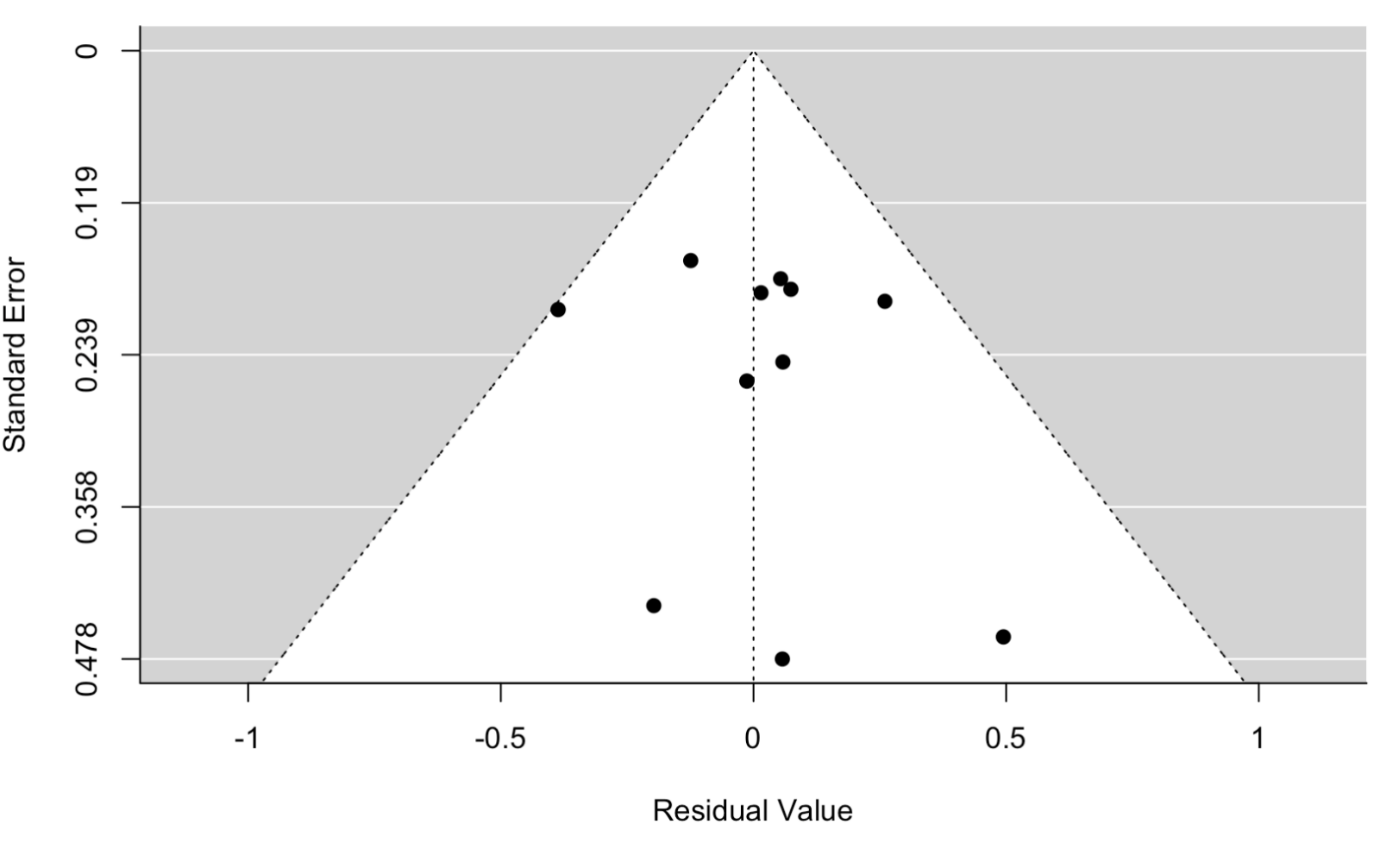
**

**Supplementary Figure S1:** Funnel plot for handedness meta-analysis in individuals with language/reading impairments corresponding to Figure 1 in the main text. Comorbid individuals are included in the language impaired group.

**
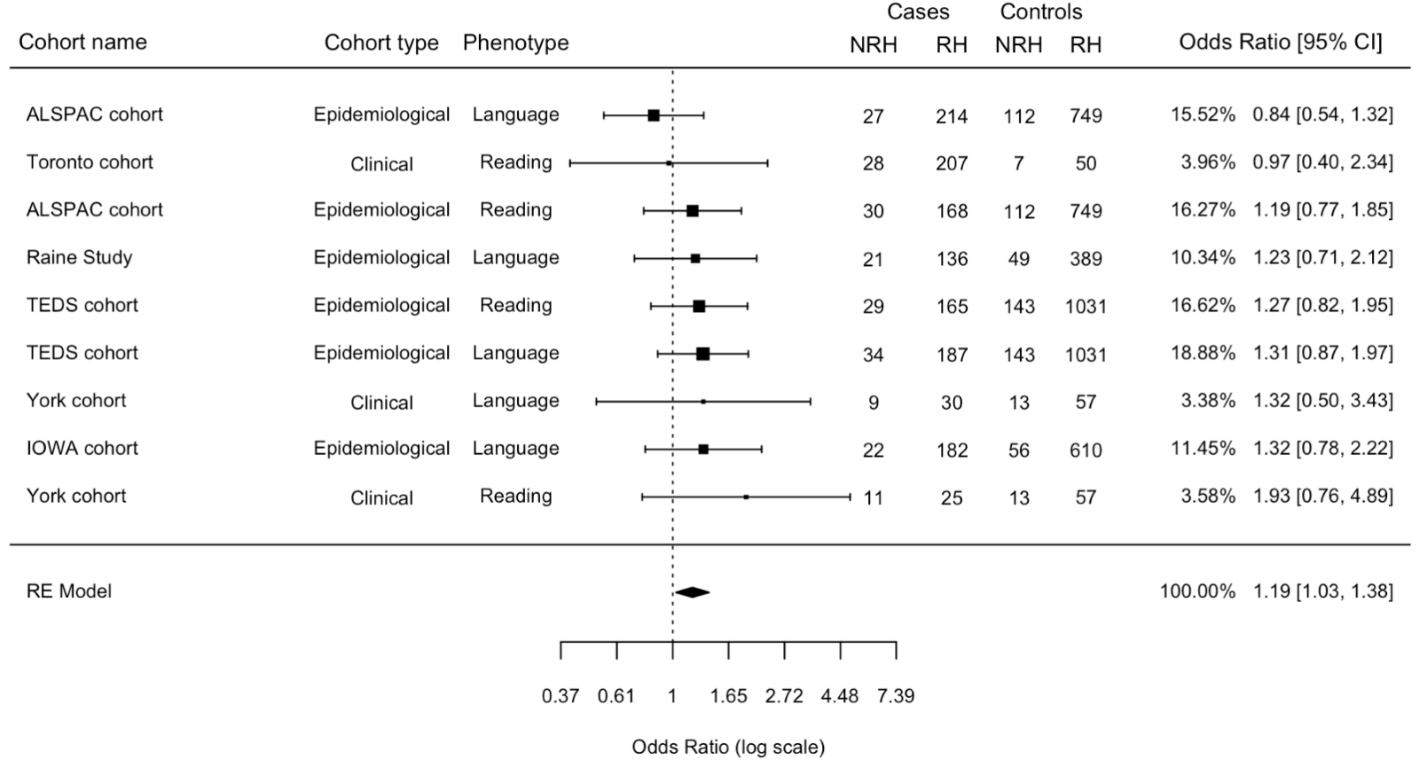
**

**Supplementary Figure S2:** Meta-analysis after excluding the Manchester Language Study and the UKDYS cohorts. Forest plot comparing the frequency of non-right handedness between individuals with reading/language impairment and controls. The forest plot contains the Odds Ratio of Random effect estimates, 95% confidence interval and the weights (in percentage) of each cohort. The meta-analysis result was OR = 1.19, CI = 1.03 - 1.38, *p* = 0.02. No heterogeneity was highlighted *(p* = 0.85)

**
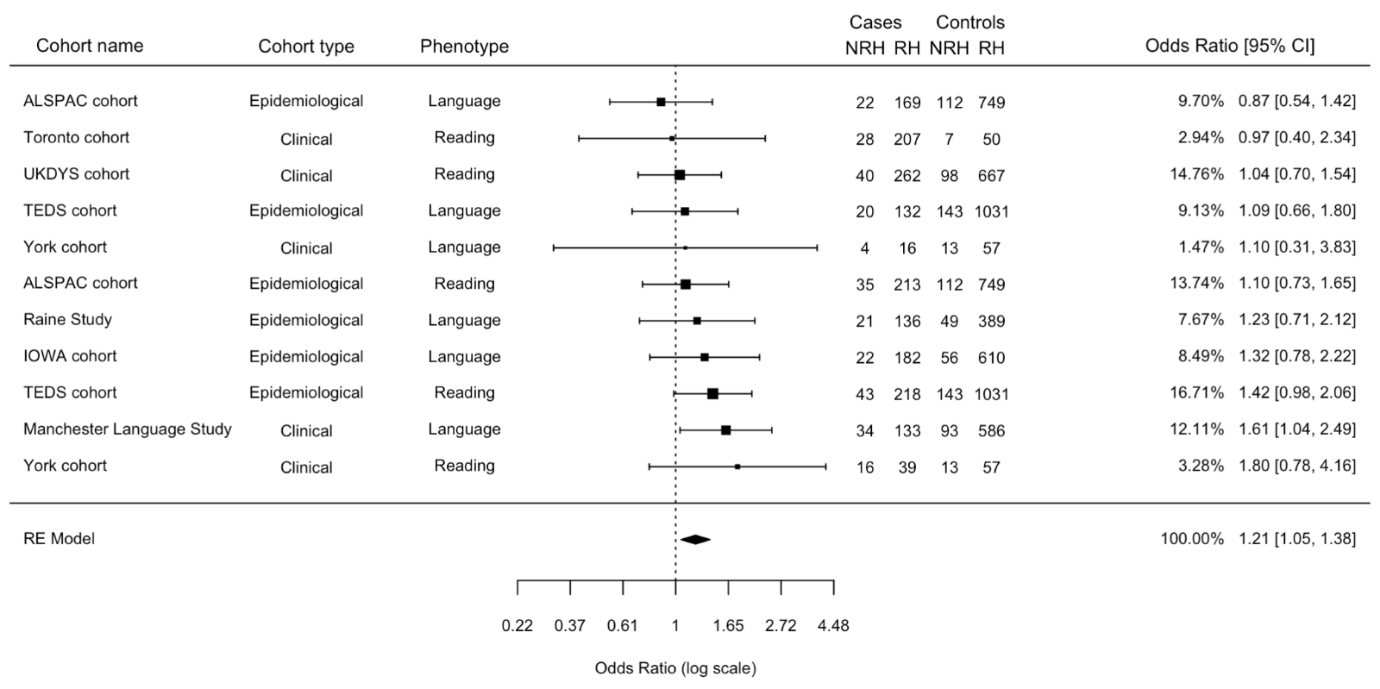
**

**Supplementary Figure S3:** Meta-analysis with comorbid individuals included in the reading impairment group. Forest plot comparing the frequency of non-right handedness between individuals with reading/language impairment and controls. The forest plot contains the Odds Ratio of Random effect estimates, 95% confidence interval and the weights (in percentage) of each cohort. The meta-analysis result was OR = 1.21, CI = 1.05 - 1.38, *p* = 0.01. No heterogeneity was highlighted *(p* = 0.79)*.* No funnel plot asymmetry was detected (*p* = 0.93). See Supplementary Figure S4 for funnel plot. Moderator analysis showed no significant effects.

**
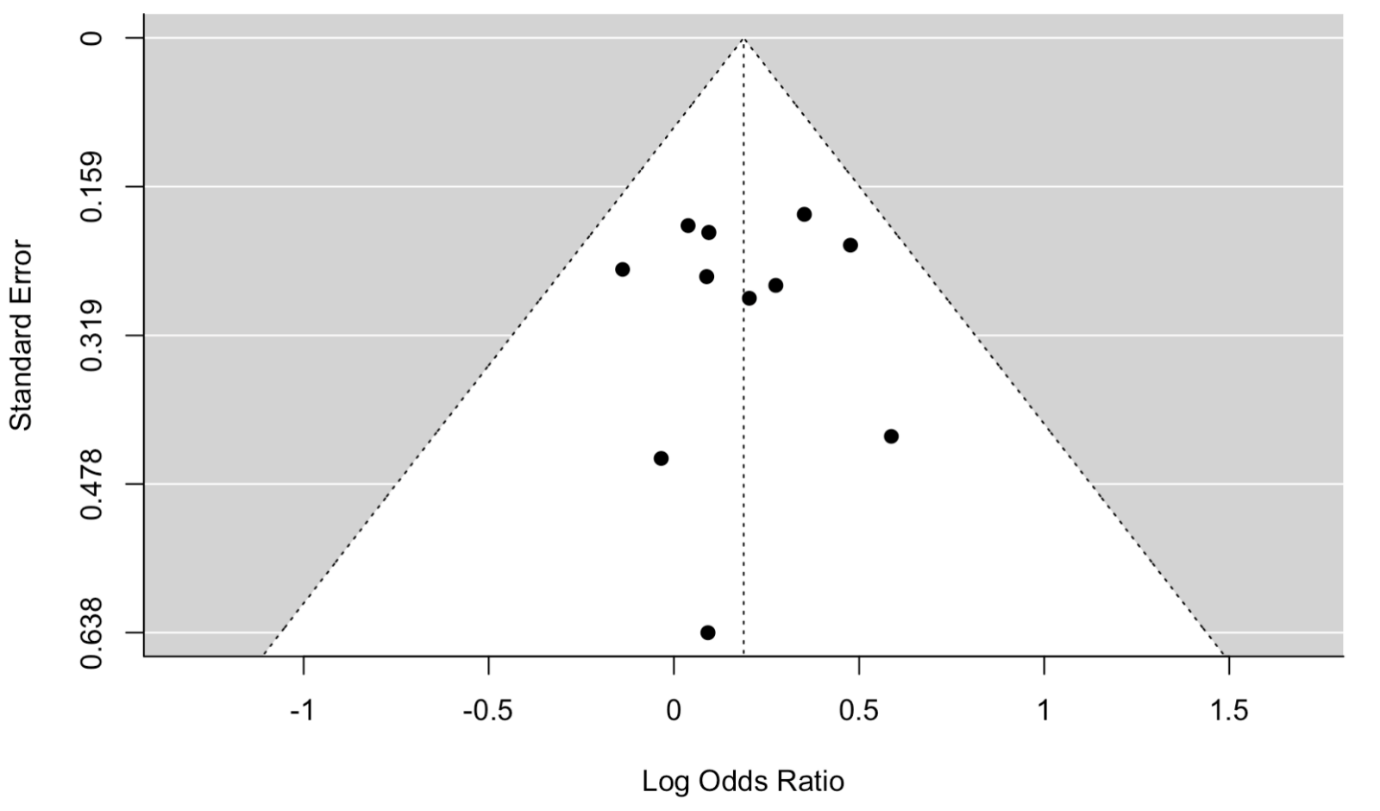
**

**Supplementary Figure S4:** Funnel plot for handedness meta-analysis corresponding to Figure S3. Comorbid individuals are included in the reading impaired group


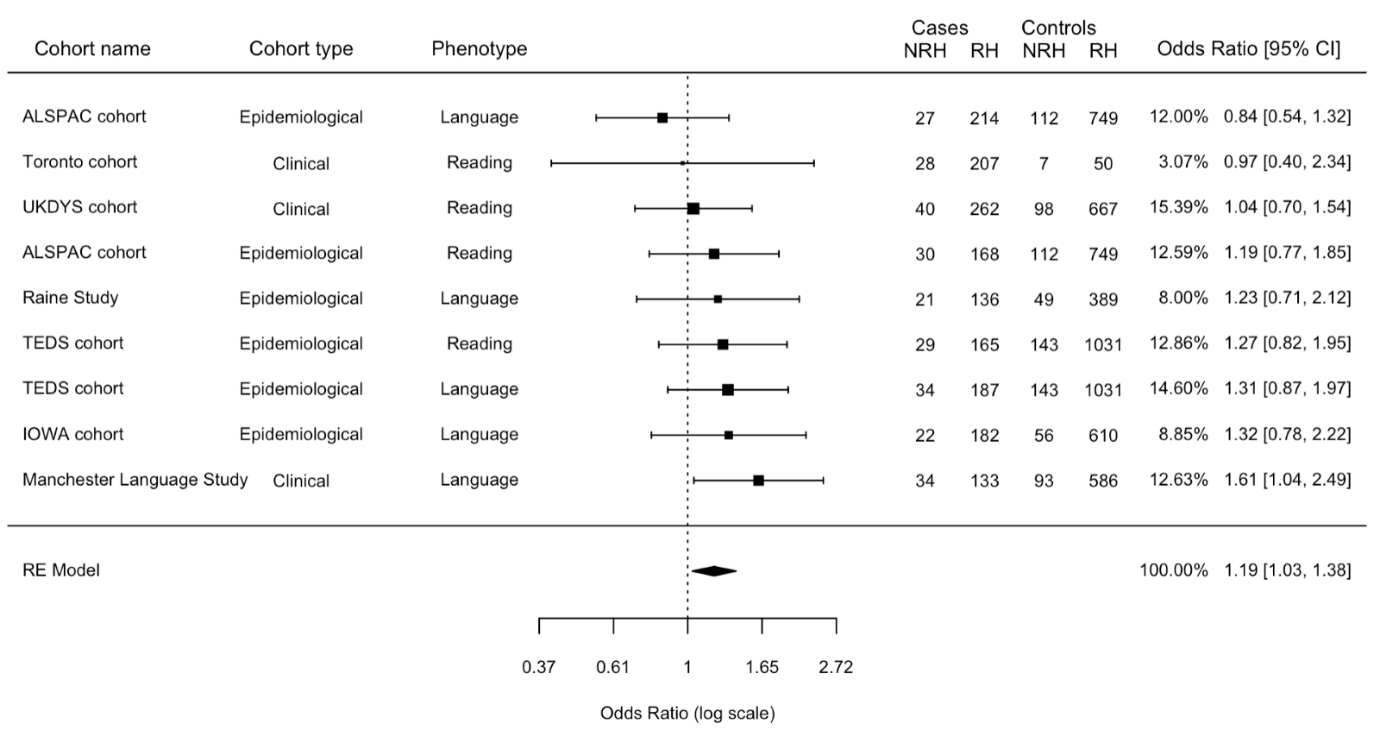


**Supplementary Figure S5:** Meta-analysis after excluding the York cohort. Forest plot comparing the frequency of non-right handedness between individuals with reading/language impairment and controls. The forest plot contains the Odds Ratio of Random effect estimates, 95% confidence interval and the weights (in percentage) of each cohort. The meta-analysis result was OR = 1.19, CI = 1.03 - 1.38, *p* = 0.02. No heterogeneity was highlighted *(p* = 0.73).

**References**

Bishop, D. V. (1998). Development of the Children’s Communication Checklist (CCC): a method for assessing qualitative aspects of communicative impairment in children. *J Child Psychol Psychiatry*, *39*(6), 879–891. Retrieved from http://www.ncbi.nlm.nih.gov/entrez/query.fcgi?cmd=Retrieve&db=PubMed&dopt=Citation&list_uids=9758196

Bishop, D. V. (2003). *Test for reception of grammar, version 2 (TROG—2)*. *Psychological Corporation*. London, UK: Psychological Corporation.

Conti-Ramsden, G., Crutchley, A., & Botting, N. (1997). The Extent to Which Psychometric Tests Differentiate Subgroups of Children With SLI. *Journal of Speech, Language, and Hearing Research*. https://doi.org/10.1044/jslhr.4004.765

Culatta, B., Page, J. L., & Ellis, J. (1983). Story Retelling as a Communicative Performance Screening Tool. *Language, Speech, and Hearing Services in Schools*. https://doi.org/10.1044/0161-1461.1402.66

Foster, H. (2007). Single word reading test 6-16. *Windsor: NFER-Nelson.*

Gathercole, S. E., Willis, C. S., Baddeley, A. D., & Emslie, H. (1994). The Children’s Test of Nonword Repetition: a test of phonological working memory. *Memory*, *2*(2), 103–127. https://doi.org/10.1080/09658219408258940

Hayiou-Thomas, M. E., Smith-Woolley, E., & Dale, P. S. (2021). Breadth versus depth: Cumulative risk model and continuous measure prediction of poor language and reading outcomes at 12. *Developmental Science*. https://doi.org/10.1111/desc.12998

Raven, J. C., Court, J. H., & Raven, J. (1996). Manual for Raven’s Standard Progressive Matrices and vocabulary scales. *1996*.

Rust, J. (1996). *WOLD Wechsler Objective Language Dimensions Manual*. London, UK: he Psychological Corporation.

Rust, J., Golombok, S., & Trickey, G. (1993). *WORD: Wechsler Objective Reading Dimensional Manual*. Sidcup, UK: Psychological Corporation.

Schulte-Körne, G., Deimel, W., Müller, K., Gutenbrunner, C., & Remschmidt, H. (1996). Familial aggregation of spelling disability. *Journal of Child Psychology and Psychiatry and Allied Disciplines*. https://doi.org/10.1111/j.1469-7610.1996.tb01477.x

Semel, E. m., Wiig, E. H., & Secord, W. (1992). *Clinical Evaluation of Language Fundamentals-Revised*. San Antonio: Psychological Corporation.

Semel, E., Wiig, E. H., & Secord, W. (1995). Clinical Evaluation of Language Fundamentals, Third Edition. *The Psychological Corporation*.

Thomson, M. E. (1982). The assessment of children with specific reading difficulties (dyslexia) using the British Ability Scales. *Br J Psychol*, *73*(Pt 4), 461–478. Retrieved from http://www.ncbi.nlm.nih.gov/entrez/query.fcgi?cmd=Retrieve&db=PubMed&dopt=Citation&list_uids=7171921

Wechsler, D. (2005). *Wechsler Individual Achievement Test (WIAT-II UK)*. *WIAT-II UK*.

Wechsler, D. (2012). Wechsler preschool and primary scale of intelligence—fourth edition. *The Psychological Corporation San Antonio, TX*.

Wechsler, D., Golombok, S., & Rust, J. (1992). *WISC-IIIUK: Wechsler Intelligence Scale for Children.* Sidcup, UK: The Psycological Corporation.

Weiß RH. (1998). Grundintelligenztest Skala 2. CFT-20 [Culture Fair Intelligence Test]. *Göttingen, Germany: Hogrefe*.

Wilkinson, G. (1993). Wide Range Achievement Test WRAT. *Wide Range Inc*.

Woodcock, R. W. (1987). *Woodcock reading mastery tests-revised*. American Guidance Service Circle Pines, MN.
